# Distinct effects of monetary versus social reward magnitudes on the emotional congruency effect in subclinical depression: a word-face Stroop study

**DOI:** 10.3389/fpsyg.2026.1767155

**Published:** 2026-03-19

**Authors:** Yang Liu, Yan Zhao, Shiping Cheng

**Affiliations:** 1 Xinjiang Key Laboratory of Mental Development and Learning Science, College of Psychology, Xinjiang Normal University, Urumqi, China; 2 School of Marxism, Xinjiang Normal University, Urumqi, China

**Keywords:** emotional congruency effect, monetary reward, reward gradient, social reward, subclinical depression, word-face Stroop task

## Abstract

**Background:**

Although incentives modulate cognitive processes, their differential effects of monetary versus social reward gradients on emotional conflict resolution in subclinical depression remain poorly understood. This study investigated how reward type and magnitude influence emotional congruency effects in this population.

**Methods:**

Two experiments used a word-face Stroop paradigm with a Latin square design. Ninety-nine undergraduates (47 with subclinical depression) completed monetary (0, 20, 100, 500 CNY) and social (no praise to university-level praise) reward tasks. A mixed-design ANOVA examined effects of reward level, emotional congruency, and group.

**Results:**

The subclinical depression group showed reduced emotional congruency, marked by lower accuracy in congruent trials than controls. Monetary rewards exhibited a threshold effect: accuracy gains plateaued beyond 100 CNY with no further improvement at 500 CNY. Social reward modulation was similar between groups, though reaction times under class-level praise were significantly higher in incongruent trials. Notably, under high monetary rewards (500 CNY), the subclinical group demonstrated shorter reaction times in congruent trials, indicating heightened sensitivity to substantial incentives.

**Conclusion:**

Emotional conflict processing in subclinical depression is differentially modulated by reward type and magnitude. Monetary rewards show saturating effects, while social rewards elicit uniform nonlinear influences. These findings highlight reward sensitivity as a crucial factor for cognitive-affective profiling and targeted interventions.

## 1 Introduction

Emotion exerts dual facilitative and inhibitory effects on cognitive processes, with emotional conflict representing a central focus in cognitive psychology research (Hu et al., 2008). Classical Stroop paradigms and their derivatives, such as the word-face Stroop task, provide essential tools for investigating the integration of multimodal information during cognitive processing (Fan et al., 2018). Although incentive mechanisms are known to modulate cognitive performance (Padmala and Pessoa, 2011), extant research primarily contrasts healthy populations with clinically diagnosed affective disorder patients, leaving a significant gap in understanding individuals with subclinical depressive tendencies.

Epidemiological studies indicate that a substantial proportion of college students worldwide experience mental health challenges. The WHO’s “World Mental Health International College Student” initiative reported that approximately 35% of first-year students across 19 universities in eight countries met criteria for at least one common psychiatric disorder (Auerbach et al., 2018). A more recent multicenter study conducted in 2022, which sampled 1,670 university students from Germany, Sweden, and Portugal, revealed alarmingly high rates of mild to severe depressive symptoms, with prevalence reaching 72.7% in Germany, 62.9% in Sweden, and 60.3% in Portuga (Pinho et al., 2025). Similarly, Report on the development of Chinese national mental health (2023–2024) revealed that depressive symptoms peak among young adults aged 18–24, with 18.5% demonstrating significant depressive tendencies and associated emotional conflict features (CAS Institute of Psychology, 2025). Although these symptoms do not meet clinical diagnostic thresholds, they substantially impair academic functioning and subjective well-being (Wongtongkam, 2019). While emerging studies have begun to characterize subclinical populations with social anxiety and trait anxiety (du Rocher and Pickering, 2024), the emotional-cognitive profiles and intervention pathways for college students with depressive tendencies remain poorly defined. This subclinical group constitutes a critical transitional stage on the mental health continuum; without timely intervention, their emotional dysregulation may escalate to clinical states (Tan et al., 2010).

### 1.1 Emotional conflict and the word-face Stroop paradigm

Emotional conflict arises when an individual experiences contradictory affective responses to competing options or situations (Zhu et al., 2010). The word-face Stroop paradigm, a derivative of the classical Emotional Stroop task, serves as a critical tool for assessing cognitive control under emotional interference by systematically manipulating stimulus congruency. This paradigm offers superior ecological validity compared to traditional tasks, as it simulates real-world scenarios requiring the simultaneous processing of facial expressions and linguistic cues (Botvinick et al., 2001). By superimposing emotionally valenced words onto congruent or incongruent facial expressions, it enables the examination of how multisource emotional information facilitates or interferes with cognitive processing (Zhu et al., 2010). Consequently, the word-face Stroop paradigm provides a vital methodological framework for studying social cognition and emotion regulation in near-naturalistic settings.

Emotional conflict is implicated in various mental health disorders, including Major Depressive Disorder (MDD) (Maunsell, 2004; Xue et al., 2017). Individuals with MDD often exhibit difficulties in emotional conflict processing, potentially linked to functional abnormalities in specific neural circuits. For example, patients with treatment-resistant depression show reduced accuracy on face-word Stroop tasks, indicating impaired conflict resolution (Xue et al., 2017). Studies demonstrate that depressed individuals typically perform worse on the word-face Stroop task than healthy controls (Bob et al., 2009), particularly under incongruent conditions (e.g., a happy face paired with a negative word), manifesting as lower accuracy and slower reaction times due to greater difficulty inhibiting task-irrelevant emotional information.

### 1.2 Subclinical depression: a critical transitional stage

Subclinical depression, also termed subsyndromal symptomatic depression, describes a depressive state in which individuals exhibit significant symptoms but do not meet the full diagnostic criteria for MDD (Kou et al., 2022). This condition occupies an intermediate position on the affective spectrum, yet its persistent symptoms are associated with substantial functional impairment. Empirical research indicates that college students with subclinical depression often lack access to systematic interventions, which may exacerbate cognitive-emotional dysregulation (e.g., attentional biases) and precipitate somatic symptoms (Ba and Wang, 2023).

Although subclinical depression and MDD represent distinct phenotypic expressions along the depression spectrum, their differences in emotional conflict processing mechanisms are not fully elucidated. MDD is characterized by persistent negative affect and marked functional impairment (Beblo and Dehn, 2019), whereas subclinical depression may reflect a stable vulnerability or personality trait (Hartlage et al., 1998). These differences manifest across emotional experience, cognitive processing, and neurobiological mechanisms. For instance, individuals with subclinical depression may exhibit features analogous to depressive personality traits, such as negative reactivity and constrained hedonic capacity, which may represent both epiphenomena of the subthreshold state and enduring traits. Research suggests that depressive symptom severity influences Stroop task performance, with more severe symptoms correlating with greater cognitive control deficits (Chen et al., 2022; Ros et al., 2023). While direct evidence on the word-face Stroop performance in subclinical depression is lacking, investigating the nuanced relationship between symptom severity and task performance could elucidate cognitive mechanisms across the depression spectrum, informing targeted interventions.

### 1.3 The role of reward modulation

Reward processing represents a critical perspective for understanding motivational deficits in depression. Individuals with MDD often exhibit reduced sensitivity to both monetary and social rewards (Ait Oumeziane et al., 2019; Nie et al., 2025), a feature known as anhedonia. Anhedonia is commonly parsed into two dissociable components: anticipatory anhedonia (the diminished capacity to look forward to and desire future rewards) and consummatory anhedonia (the reduced ability to experience pleasure in the moment). Notably, deficits in anticipatory pleasure may be particularly prominent in depression (Rutherford et al., 2023; Guo et al., 2023). Notably, such alterations may extend to subclinical populations, suggesting transdiagnostic significance (Liu et al., 2015). Although depressive symptoms dampen reward sensitivity, incentives may still modulate task performance. For example, appropriate motivation can enhance effort expenditure in depressed individuals (Klawohn et al., 2022). However, traditional paradigms like the incentive delay task may have limitations in distinguishing between monetary and social reward processing (Sankar et al., 2019). Recent studies incorporating reward magnitude variations reveal that individuals with subclinical depression exhibit blunted reward sensitivity under high-probability conditions, with no difference between reward types, implying that reward magnitude may be a more sensitive parameter than reward type for detecting deficits (Ba and Wang, 2023). Investigations into reward magnitude effects have yielded complex patterns, including linear relationships (Rosell-Negre et al., 2017), inverted U-shaped curves (Bijleveld et al., 2023), and evidence suggesting that social rewards may sometimes exert stronger motivational effects than monetary rewards (Wang et al., 2017). Therefore, implementing ecologically valid reward gradients (e.g., multi-level monetary/social rewards) could precisely delineate reward sensitivity in subclinical depression, uncovering distinct mechanisms.

Research on reward processing in depression has extensively examined sensitivity to reward magnitude—the objective value or size of a reward (e.g., large vs. small monetary incentives). However, a complementary line of inquiry highlights impairments in reward efficacy—the subjective valuation of a reward’s worth and the efficiency with which that value translates into motivated behavior (Shenhav, 2024). For instance, even when reward magnitudes are perceptible, individuals with depression may exhibit blunted neural and behavioral responses to reward-predicting cues, suggesting a deficit in the ability to generate motivation from anticipated rewards. This is consistent with findings that adolescents with major depression show impairments in episodic future thinking and anticipatory pleasure, which are closely tied to the constructive valuation of future rewards (Lakshmi et al., 2023).

### 1.4 Present study

The present study primarily manipulates reward magnitude (both monetary and social) to investigate how this objective dimension modulates cognitive conflict processing in subclinical depression. While not directly measuring reward efficacy, our design acknowledges that the observed effects of magnitude may be modulated by underlying differences in how rewards are subjectively valued—a critical avenue for future research.

Building directly on the theory of deficits in anticipatory anhedonia, the present study aims to dissect how distinct types of rewards—monetary (extrinsic) versus social (intrinsically valenced)—differentially modulate emotional conflict processing in subclinical depression. By systematically manipulating reward gradients within a validated emotional conflict paradigm, we can directly test for specific impairments in the motivational system. Crucially, comparing the modulatory effects of monetary and social rewards allows us to investigate whether potential deficits are generalized across reward domains or are particularly pronounced in social motivation processing—a distinction with significant theoretical implications for understanding the heterogeneity of anhedonia. This approach not only refines the cognitive-affective profile of subclinical depression but also provides a more nuanced framework than examining either reward type in isolation.

To this end, this study aims to investigate the emotional congruency effect and the modulatory role of reward magnitude in college students with subclinical depression using the word-face Stroop paradigm. Two experiments will employ a Latin square design to compare monetary (Experiment 1) and social reward (Experiment 2) gradients, each comprising four levels (0, 20, 100, 500 CNY for monetary; no praise to university-level praise for social). This design allows direct comparison of behavioral responses (reaction time and accuracy) while controlling for practice effects. By examining how varying reward magnitudes influence emotional conflict processing, this research seeks to clarify the cognitive-affective mechanisms in subclinical depression and contribute to the development of finely tuned interventions.

Based on the theory of deficits in anticipatory anhedonia, we hypothesize that:

*H1*: Compared to the healthy control group, individuals with subclinical depression will exhibit a reduced emotional congruency effect in emotional conflict processing.

*H2*: The facilitative effects of monetary rewards on behavioral performance in the subclinical depression group will demonstrate a threshold effect, such that beyond a certain moderate magnitude, higher rewards will not yield additional gains.

*H3*: The modulatory patterns of social rewards on emotional conflict processing will be relatively similar between the two groups; however, the subclinical depression group may show heightened sensitivity to specific levels of social evaluation.

## 2 Experiment 1

### 2.1 Methods

#### 2.1.1 Participants

Based on established screening criteria for non-clinical populations (Beck et al., 1996; Yang and Xiong, 2016; Zhang et al., 2020), the subclinical depression group comprised individuals with BDI-II scores ≥15 and SDS index scores >0.5, whereas the healthy control group consisted of individuals with BDI-II scores ≤14 and SDS index scores <0.5. A BDI-II score ≥15 is widely used to indicate the presence of mild to moderate depressive symptoms in community and student samples. It is important to note that a formal diagnosis of Major Depressive Disorder requires a structured clinical interview (e.g., SCID) based on full DSM-5 or ICD-11 criteria. The current study aimed to explore the symptom continuum; therefore, formal clinical diagnosis was not performed, and the term “subclinical depression” specifically refers to individuals exhibiting significant symptomatology via questionnaire measures without a confirmed clinical diagnosis. Following a rigorous screening process, a final valid sample of 110 participants was selected, with 55 individuals assigned to each group (subclinical depression and healthy control); gender distribution was balanced across both groups. To counterbalance potential order effects, participants were randomly assigned to one of two experimental sequences using a Latin square design. Group A (*n* = 55, including 27 individuals with subclinical depression) first completed the monetary reward task (Experiment 1), followed by the social reward task (Experiment 2) after a one-week interval. Group B (*n* = 55, including 28 individuals with subclinical depression) completed the tasks in the reverse order. This design ensured that the task order variable (monetary-social vs. social-monetary) was balanced across all experimental conditions, thereby minimizing confounding effects due to practice, fatigue, or transfer. The one-week inter-session interval was implemented to reduce residual carryover effects (e.g., emotional priming or response strategy inertia) from the first task, ensuring that each experiment was conducted under relatively independent conditions.

An *a priori* power analysis was conducted using G*Power software for a 2 × 2 × 4 repeated-measures analysis of variance (ANOVA). With a significance level (*α*) of 0.05, an effect size (f) of 0.25 $(\eta_{p}^{2}$ = 0.06), and a statistical power of 0.80, the analysis indicated a minimum sample size of 24 participants. After excluding outliers beyond three standard deviations from the mean in reaction times or accuracy on the word-face Stroop task, data from 99 participants were retained for the final analysis. The final sample included 47 individuals in the subclinical depression group (24 males; mean age = 18.63 ± 1.44 years) and 52 in the healthy control group (25 males; mean age = 18.88 ± 1.07 years). All participants reported normal visual and auditory function, were right-handed, had no history of neurological or psychiatric disorders, and provided voluntary informed consent before receiving corresponding compensation.

#### 2.1.2 Experimental apparatus and materials

Stimulus presentation was controlled by an HP Pavilion Laptop 15 with a screen resolution of 1920 × 1080. All visual stimuli were edited and standardized using Adobe Photoshop CS3. Experimental materials comprised 80 facial expressions selected from the standardized Chinese Facial Affective Picture System (Gong et al., 2011), all with high ecological validity (recognition rate > 90%). The experiment began with a block of 32 practice trials, followed by the formal test comprising a total of 256 trials. Within the formal trials, congruent (word–expression match) and incongruent (mismatch) conditions were equally represented, with 128 trials each. Positive (happy) and negative (sad) expressions were equally represented (50% each) and gender ratio was strictly balanced (1:1). Emotional words (“happy” or “sad”) were superimposed onto the central region of each face to create the congruent and incongruent conditions. Participants were instructed to ignore word semantics and judge the facial expression valence. All stimuli were converted to grayscale to enhance emotional interference. A pseudo-randomized sequence (maximum two consecutive trials of the same type) was implemented via E-Prime 2.0 to control for order effects.

#### 2.1.3 Experimental design

A 4 (monetary reward: 0, 20, 100, 500 CNY) × 2 (emotional condtion: congruent, incongruent) × 2 (group: subclinical depression, healthy control) mixed-design was employed. The dependent variables were reaction time and accuracy. The group factor was a between-subjects variable, whereas reward level and emotional condition were treated as within-subjects factors. This design enables robust examination of main and interaction effects among reward magnitude, emotional conflict, and group membership on cognitive-affective processing.

#### 2.1.4 Experimental procedure

**Figure 1:**
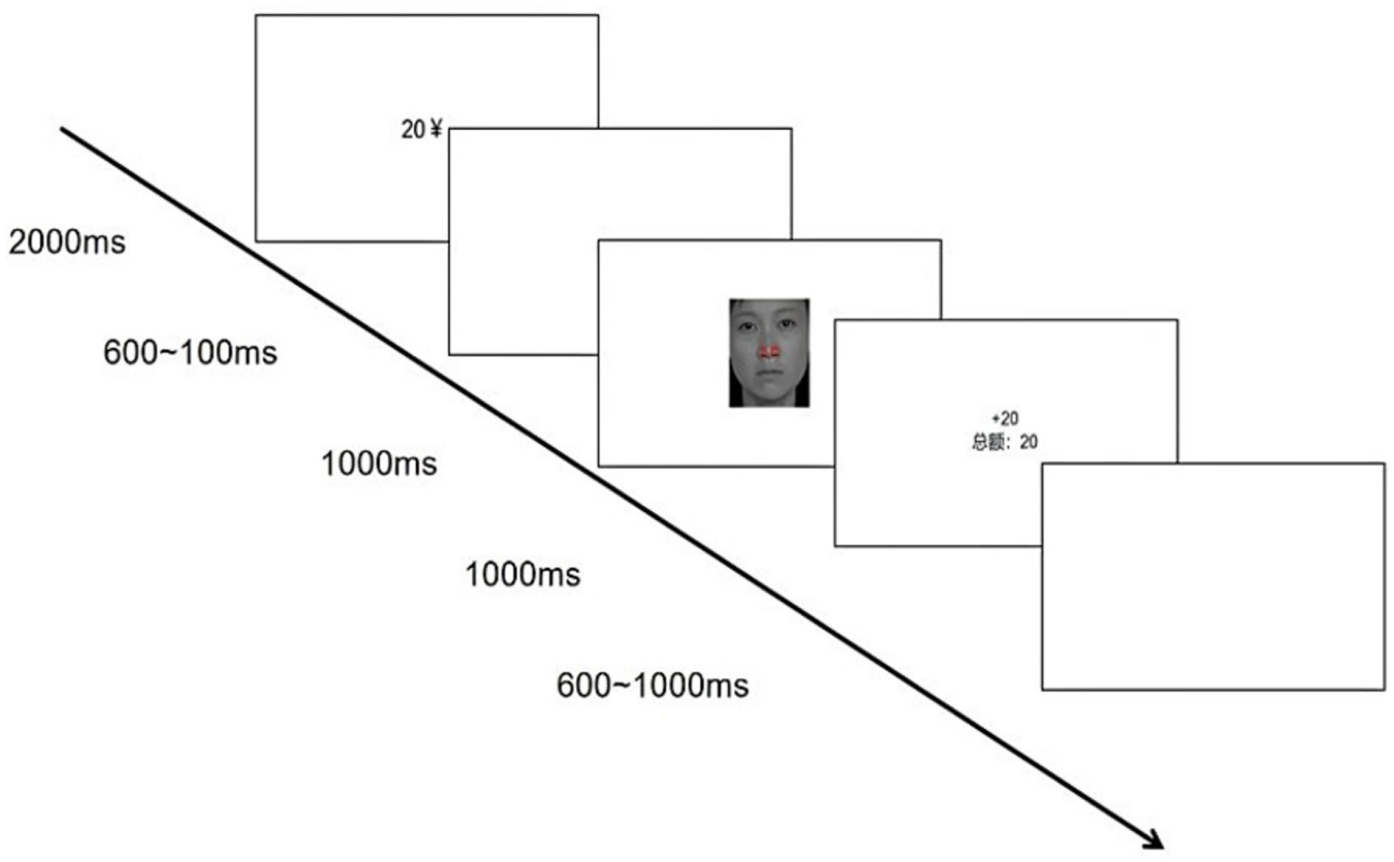
Trial sequence in the monetary reward condition. Facial images reproduced from Gong et al. (2011) with permission from the Chinese Facial Affective Picture System (CFAPS) by Shenzhen Brain & Intelligence Development Technology Co., Ltd., under the terms of the AGREEMENT FOR THE USE OF THE CHINESE EMOTIONAL STIMULI MATERIALS DATABASE. Source: Shenzhen Brain & Intelligence Development Technology Co., Ltd.

Participants first completed a 32-trial practice block. The formal task consisted of two blocks (128 trials each, 256 total), with self-paced breaks between blocks. Each trial followed this sequence: a reward cue (amount + ¥ symbol) for 2000 ms; a 600–1,000 ms blank screen; a 1,000 ms target (word–face compound); performance feedback (displayed until response); and a final 600–1,000 ms blank screen. Response mappings were fixed: “F” for positive and “J” for negative expressions. After practice, participants could proceed or repeat based on self-assessed readiness. For reward trials, correct responses triggered gain feedback (“+20” to “+500”), while errors showed “0,” with cumulative earnings displayed throughout. This design ensured explicit incentive salience and ecological engagement (see Figure 1).

### 2.2 Research results

A repeated-measures ANOVA on reaction time data revealed a significant main effect of reward level (see Tables 1, 2). *Post hoc* tests (LSD) indicated that reaction times under the 500 CNY condition were significantly shorter than those under both the 0 CNY condition (*p* < 0.01) and the 20 CNY condition (*p* < 0.05). No significant difference was observed between the 500 CNY and 100 CNY conditions (*p* > 0.05), and reaction times among the 0, 20, and 100 CNY conditions did not differ significantly.

**Table 1:** Mean reaction times and accuracy (M ± SD) for the subclinical depression group and healthy control group.

Group	Emotional condition	0 CNY	20 CNY	100 CNY	500 CNY
Reaction times					
Subclinical depression	Incongruent	676.15 ± 92.06	692.31 ± 103.38	681.98 ± 95.38	674.34 ± 114.34
	Congruent	635.95 ± 84.09	627.53 ± 76.10	617.90 ± 86.27	620.70 ± 91.06
Healthy control	Incongruent	696.73 ± 100.14	679.58 ± 116.10	688.16 ± 105.58	664.10 ± 109.10
	Congruent	639.73 ± 92.41	635.34 ± 93.62	630.55 ± 99.47	625.55 ± 100.62
Accuracy					
Subclinical depression	Incongruent	0.91 ± 0.05	0.92 ± 0.07	0.93 ± 0.08	0.93 ± 0.09
	Congruent	0.96 ± 0.06	0.96 ± 0.05	0.98 ± 0.03	0.98 ± 0.04
Healthy control	Incongruent	0.90 ± 0.10	0.92 ± 0.08	0.91 ± 0.10	0.93 ± 0.07
	Congruent	0.98 ± 0.04	0.98 ± 0.04	0.98 ± 0.03	0.98 ± 0.04

**Table 2:** ANOVA for reaction times in the subclinical depression and healthy control groups.

Source of variation	*df*	*MS*	*F*	*p*	η_p_^2^
Monetary reward (MR)	3	9197.80	5.06	0.002	0.096
Emotional condition (EC)	1	549466.18	196.08	0.000	0.667
Group	1	197.76	0.03	0.875	0.000
MR* Group	3	3026.83	1.67	0.175	0.037
EC * Group	1	1995.78	0.71	0.401	0.006
MR * EC	3	2085.95	1.23	0.301	0.003
MR * EC * Group	3	3409.75	2.00	0.114	0.054

A significant main effect of emotional condition was also found. Participants responded significantly faster in the congruent condition than in the incongruent condition (*p* < 0.01). No interaction effects reached significance.

For accuracy data, the ANOVA showed a significant main effect of monetary reward (see Table 3). Post hoc tests demonstrated that accuracy under the 500 CNY condition was significantly higher than under both the 0 CNY (*p* < 0.01) and 20 CNY conditions (*p* < 0.05), with no significant difference compared to the 100 CNY condition (*p* > 0.05). A significant main effect of emotional condition was also identified. Accuracy was significantly higher in the congruent condition than in the incongruent condition (*p* < 0.01). No significant interactions were observed.

**Table 3:** ANOVA for accuracy rates in the subclinical depression and healthy control groups.

Source of variation	*df*	*MS*	*F*	*p*	η_p_^2^
MR	3	0.01	3.53	0.015	0.035
EC	1	0.65	104.51	0.000	0.519
Group	1	1.01	0.00	0.993	0.000
MR* Group	3	0.00	0.94	0.424	0.002
EC * Group	1	0.02	2.59	0.111	0.025
MR * EC	3	0.00	0.85	0.469	0.020
MR * EC * Group	3	0.01	1.73	0.162	0.041

To address a potential confounding effect of gender, an exploratory four-way mixed-design ANOVA was conducted on the primary dependent variables (reaction time and accuracy), with Gender (male, female) added as an additional between-subjects factor to the original model. This analysis examined all possible interactions involving Gender, Group, Reward Level, and Emotional Condition. Results revealed no significant main effect of Gender on either reaction time (*F* < 1) or accuracy [*F*(1, 95) = 0.42, *p* = 0.52, ηp^2^ = 0.004]. Furthermore, no significant two-way, three-way, or four-way interactions involving Gender were found (all *ps*> 0.15). These exploratory results suggest that gender did not significantly moderate or confound the key effects of monetary reward gradients on emotional conflict processing reported in the primary analyses.

In summary, Experiment 1 demonstrated that monetary reward magnitude modulated task performance in a threshold-dependent manner. Specifically, the highest reward (500 CNY) facilitated faster responses and higher accuracy compared to lower or no-reward conditions, with performance gains saturating beyond 100 CNY. Furthermore, a robust emotional congruency effect was observed across all reward levels, indicating universally faster and more accurate responses in congruent trials. These findings suggest that while high-magnitude monetary incentives can enhance both the speed and precision of emotional conflict processing, this facilitative effect is subject to a nonlinear saturation point.

## 3 Experiment 2

### 3.1 Methods

#### 3.1.1 Participants

The participant cohort in Experiment 2 was identical to that described in Experiment 1. After excluding outliers beyond three standard deviations from the mean in reaction time or accuracy on the word-face Stroop task, data from 93 participants were retained for analysis. The final sample consisted of 44 undergraduates with subclinical depression (21 males; mean age = 18.94 ± 1.56 years) and 49 healthy controls (23 males; mean age = 18.36 ± 1.17 years). All procedural details, including task administration and environmental conditions, were consistent with Experiment 1.

#### 3.1.2 Experimental apparatus and materials

The apparatus and experimental setup remained unchanged from Experiment 1. The key modification involved replacing monetary reward cues with social reward images, which depicted varying levels of social approval (no praise, class-, school-, and university-level praise). All other stimulus parameters-including facial expression stimuli, word-face compound design, and grayscale processing-were identical to those previously described.

The social reward images used in this study were selected and standardized through a pre-experimental validation procedure. Specifically, a set of candidate images depicting positive social interactions (e.g., smiling, applause, approval gestures) was compiled. These images, along with a set of neutral control images (e.g., household objects), were then rated by an independent sample of 20 participants (who did not partake in the main experiments) on three 9-point Likert scales: valence (1 = very unpleasant to 9 = very pleasant), arousal (1 = very calming to 9 = very exciting), and social relevance (1 = not at all social to 9 = highly social). As intended, the social reward images were rated significantly higher than the neutral images on both valence [t(19) = 8.84, *p* < 0.001, *d* = 1.03] and social relevance [t(19) = 12.14, *p* < 0.001, *d* = 1.15]. The final set of images was chosen based on these ratings to ensure they were high in positive valence and perceived social content.

#### 3.1.3 Experimental design

A 4 (social reward: no praise, class-, school-, university-level praise) × 2 (emotional condition: congruent, incongruent) × 2 (group: subclinical depression, healthy control) mixed-design was employed. The dependent variables were reaction time and accuracy.

#### 3.1.4 Experimental procedure

**Figure 2:**
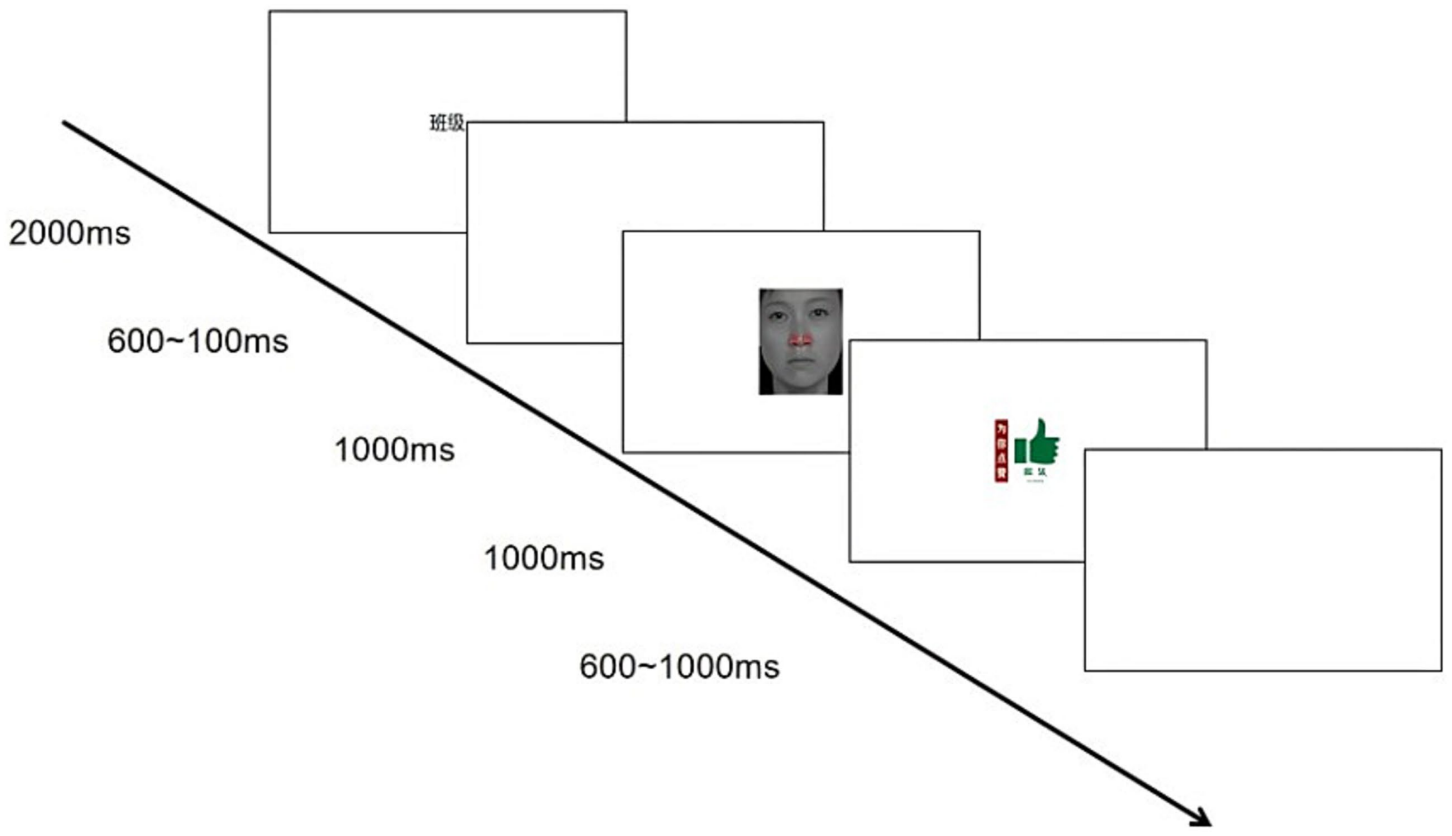
Trial sequence in the social reward condition. Facial images reproduced from Gong et al. (2011) with permission from the Chinese Facial Affective Picture System (CFAPS) by Shenzhen Brain & Intelligence Development Technology Co., Ltd., under the terms of the AGREEMENT FOR THE USE OF THE CHINESE EMOTIONAL STIMULI MATERIALS DATABASE. Source: Shenzhen Brain & Intelligence Development Technology Co., Ltd.

The procedure mirrored that of Experiment 1, with the exception that social reward cues were substituted for monetary incentives (see Figure 2).

### 3.2 Research results

A repeated-measures ANOVA on reaction time data revealed no significant main effect of reward level (see Tables 4, 5). However, a significant main effect of emotional condition was observed with faster responses in congruent trials. The main effect of group was not significant.

**Table 4:** Mean reaction times and accuracy (M ± SD) for the subclinical depression group and healthy control group.

Group	Emotional condition	No praise	Class-level praise	Class-level praise	University-level praise
Reaction times					
Subclinical depression	Incongruent	639.64 ± 110.59	649.51 ± 108.32	632.10 ± 91.91	636.80 ± 114.63
	Congruent	600.36 ± 90.20	605.62 ± 87.04	611.45 ± 106.89	624.97 ± 100.62
Healthy control	Incongruent	622.78 ± 82.81	633.01 ± 84.36	618.38 ± 88.11	620.71 ± 79.34
	Congruent	574.62 ± 77.04	567.92 ± 83.32	577.17 ± 78.32	579.04 ± 77.08
Accuracy					
Subclinical depression	Incongruent	0.90 ± 0.09	0.89 ± 0.08	0.90 ± 0.10	0.91 ± 0.08
	Congruent	0.94 ± 0.07	0.94 ± 0.06	0.94 ± 0.06	0.92 ± 0.08
Healthy control	Incongruent	0.89 ± 0.09	0.89 ± 0.09	0.89 ± 0.10	0.89 ± 0.08
	Congruent	0.95 ± 0.06	0.97 ± 0.05	0.95 ± 0.06	0.95 ± 0.07

**Table 5:** ANOVA for reaction times in the subclinical depression and healthy control groups.

Source of variation	*df*	*MS*	*F*	*p*	η_p_^2^
Social reward (SR)	3	1673.02	1.06	0.368	0.012
EC	1	278293.87	74.97	0.000	0.454
Group	1	15305.03	2.37	0.127	0.026
SR* Group	3	797.74	0.50	0.680	0.006
EC * Group	1	18547.35	4.10	0.028	0.053
SR * EC	3	7287.96	3.23	0.023	0.035
SR * EC * Group	3	848.38	0.38	0.771	0.004

Critically, a significant interaction emerged between emotional condition and reward level. Simple effect analyses showed that under the incongruent condition, reward level had no significant effect, *F* (3, 90) = 1.81, *p* > 0.05, η_p_^2^ = 0.008. However, pairwise comparisons indicated that reaction times under class-level praise were significantly higher than under no praise, school-level, or university-level praise (*ps*< 0.05), with no differences among the latter three. Under the congruent condition, reward level significantly influenced performance, *F* (3, 90) = 2.87, *p* < 0.05, η_p_^2^ = 0.089. Reaction times under university-level praise were significantly higher than under no praise (*p* < 0.01) and class-level praise (*p* < 0.01), but not school-level praise (*p* > 0.05).

For accuracy data, no significant main effects were found for social reward level or group (see Table 6). A significant main effect of emotional condition was observed with higher accuracy in congruent trials. A key interaction between emotional condition and group was detected. Simple effects analysis revealed no group difference under the incongruent condition (*p* > 0.05). However, under the congruent condition, the healthy control group demonstrated significantly higher accuracy than the subclinical depression group, *F* (1, 90) = 7.86, *p* < 0.01, η_p_^2^ = 0.080. Within-group comparisons showed that both groups had higher accuracy in congruent trials (*ps*< 0.01).

**Table 6:** ANOVA for accuracy rates in the subclinical depression and healthy control groups.

Source of variation	*df*	*MS*	*F*	*p*	η_p_^2^
Social reward (SR)	3	0.00	0.20	0.894	0.002
EC	1	0.44	64.35	0.000	0.417
Group	1	0.00	0.51	0.478	0.998
SR* Group	3	0.00	0.35	0.792	0.004
EC * Group	1	0.05	7.42	0.008	0.076
SR * EC	3	0.01	1.33	0.264	0.015
SR * EC * Group	3	0.00	0.43	0.734	0.005

A parallel exploratory four-way mixed-design ANOVA was performed for the social reward experiment, incorporating Gender as an additional between-subjects factor. The analysis indicated no significant main effect of Gender on reaction time [*F*(1, 89) = 0.89, *p* = 0.35, ηp^2^ = 0.010] or accuracy (*F* < 1). Critically, none of the interactions involving Gender reached statistical significance (all *ps*> 0.10), including the Gender × Group and Gender × Group × Emotional Condition interactions. This pattern of null findings indicates that the modulatory effects of social reward levels and the observed group differences in emotional conflict processing were not significantly influenced by participant gender in this study.

In summary, Experiment 2 revealed that social rewards modulated emotional conflict processing in a context-dependent manner, with key interactions involving reward level and group. Unlike monetary rewards, social praise did not produce a uniform main effect but interacted with emotional context: class-level praise uniquely slowed responses in incongruent trials, whereas university-level praise slowed responses specifically in congruent trials. Critically, group differences emerged only in accuracy during congruent trials, where the subclinical depression group performed worse than controls. This pattern indicates that social evaluation exerts a more complex, situation-specific influence on performance, and highlights a specific deficit in processing congruent social–emotional cues within the subclinical depression group.

## 4 Discussion

The present study investigated the differential effects of monetary versus social rewards on emotional conflict processing in college students with subclinical depression using a word-face Stroop paradigm. Our findings reveal distinct modulation patterns: while both reward types influenced performance in the subclinical depression group, monetary rewards primarily enhanced response accuracy under high-magnitude conditions following a threshold pattern, whereas social rewards elicited a more uniform, non-linear modulation on reaction times across groups. These results highlight the multifaceted nature of reward processing in subclinical depression and provide insights into potential intervention strategies.

### 4.1 Altered emotional conflict processing in subclinical depression

Students with subclinical depression exhibited a stable emotional congruency effect, demonstrating the characteristic performance degradation during incongruent trials observed in healthy populations. When confronted with incongruent emotional information, individuals showed markedly slower responses and reduced accuracy, reflecting the cognitive process of inhibiting task-irrelevant stimuli (Başgöze et al., 2015). This finding aligns with previous research by Haas et al. (2006), Etkin et al. (2006), and Xue et al. (2017), further validating the universality of the emotional congruency effect. The emotional congruency effect underscores that cognitive processing is facilitated when emotional information aligns with task demands, leading to faster and more accurate responses. Conversely, when emotional conflicts arise, reaction times prolong and accuracy declines, highlighting the disruptive impact of emotional interference on cognitive efficiency. This emphasizes the importance of maintaining consistency between emotional cues and task requirements to optimize cognitive performance during complex emotional information processing.

Further analysis revealed that college students with subclinical depression and their healthy counterparts exhibited distinct emotional congruency effects during the word-face Stroop task. A significant group difference was observed specifically under the congruent condition, where the healthy control group demonstrated significantly higher accuracy than the subclinical depression group. In contrast, no significant performance difference was found between the two groups under the incongruent condition. This pattern suggests a specific impairment in emotional conflict processing among individuals with depressive tendencies. Existing literature provides potential mechanisms for these findings. Healthy individuals typically exhibit slowed reaction times when processing incongruent information, reflecting their capacity to inhibit task-irrelevant stimuli (Başgöze et al., 2015). Conversely, Studies have found that MDD patients typically display an attentional bias toward negative emotional information during Stroop tasks, which may lead to increased interference effects in the word-face Stroop paradigm (Guo et al., 2018). Furthermore, alterations in cognitive and emotional processing may persist even during remission periods in MDD (Fang et al., 2022). Neuroimaging studies indicate that individuals with depression may show altered activation in the anterior cingulate cortex (ACC) during emotional Stroop tasks (Loeffler et al., 2019), potentially explaining their differential performance across congruency conditions. Furthermore, individuals with depressive tendencies often demonstrate an attentional bias toward negative stimuli (Wingenfeld et al., 2011), which may diminish the typical advantage observed in congruent conditions by amplifying interference from negative emotional content regardless of congruency. Notably, within the monetary reward task of the current study, the subclinical depression group continued to show lower accuracy than the healthy controls under congruent conditions. This persistent deficit suggests that depressive tendencies may exert long-term negative effects on cognitive processing. Even under the modulation of external reward incentives, these inherent cognitive impairments may not be fully compensated, highlighting the profound and enduring nature of cognitive dysfunction associated with subclinical depression.

### 4.2 Monetary reward modulation: threshold effects and neural mechanisms

This study revealed that the influence of monetary rewards on college students with subclinical depression is not a simple “more is better” relationship, but rather a complex nonlinear one. Specifically, the findings showed that the reaction times of students with subclinical depression on emotionally congruent tasks were significantly shorter than those of healthy controls, indicating that appropriate monetary incentives can enhance the effort level of these individuals (Jia et al., 2021). This difference was particularly pronounced under the 100 CNY and 500 CNY reward conditions. This suggests that monetary rewards of a relatively higher magnitude may possess a powerful motivational effect, effectively capturing the attention of students with subclinical depression and thereby optimizing their performance on congruent trials, further supporting the positive modulatory role of monetary rewards on their cognitive processing.

A key finding was the presence of a distinct threshold characteristic in the incentive effects of monetary rewards. When the reward amount increased from low levels (0 CNY, 20 CNY) to medium and high levels (100 CNY, 500 CNY), the behavioral performance of the subclinical depression group showed significant improvement—reaction times under the 500 CNY condition were significantly shorter than those under the 0 CNY and 20 CNY conditions, and accuracy under both the 100 CNY and 500 CNY conditions was significantly higher than under the 0 CNY and 20 CNY conditions. However, no statistically significant differences were found in either accuracy or reaction time between the 100 CNY and 500 CNY reward conditions. This indicates that once the reward amount reaches a certain critical point (e.g., 100 CNY), its incentive effect tends to saturate and does not increase linearly with further increases in the monetary amount.

From a cognitive-neurobiological perspective, this nonlinear relationship may be closely linked to the core feature of a blunted reward system function in individuals with subclinical depression. Patients with MDD frequently exhibit reduced neural reactivity to reward stimuli, indicative of decreased reward sensitivity (Toobaei et al., 2025). A relatively low monetary amount, such as 20 CNY, may possess an incentive salience that is insufficient to surpass the activation threshold of their compromised reward system, thereby failing to effectively mobilize effort. In contrast, when the reward amount reaches a level such as 100 CNY or 500 CNY, the stimulus intensity may be adequate to activate their reward circuitry, leading to subsequent improvements in behavioral performance. However, once this “activation threshold” is exceeded, further increases in the monetary amount fail to produce a linear enhancement in performance, likely due to constraints in fundamental cognitive resources or neural responsiveness. This mechanistic account explains the absence of a significant difference in effects between the 100 CNY and 500 CNY conditions.

### 4.3 Social reward modulation: normative patterns and cultural specificity

The study found that the modulatory effect of social reward levels on emotional conflict in college students with subclinical depression was consistent with that observed in healthy controls. Cognitive control refers to an individual’s ability to regulate their thoughts and behaviors. Research has consistently demonstrated that this capacity is impaired in individuals with MDD, which may underlie their poorer performance on tasks requiring the inhibition of interfering information, such as incongruent emotional trials (Chen et al., 2022). However, the finding that the performance of the subclinical depression group on the incongruent task did not significantly differ from that of the healthy control group in the present social reward context suggests that their inherent cognitive control deficits might have been compensated for by other factors. Social rewards are a plausible candidate for such a compensatory mechanism. The motivational salience of social approval may enhance engagement and resource allocation, potentially normalizing behavioral outputs despite underlying neurocognitive differences. In this study, it is likely that the modulatory influence of social rewards contributed to the consistent behavioral patterns observed between students with subclinical depression and healthy controls across both emotional conflict and congruency situations. This alignment in performance underscores the potential of socially meaningful incentives to engage motivational circuits and facilitate adaptive cognitive-emotional functioning even in the presence of subclinical depressive symptoms.

Social reward, as a form of external incentive, may influence emotional conflict through multiple mechanisms. Firstly, receiving social rewards (e.g., recognition, praise, or support) can enhance an individual’s self-esteem and social self-efficacy (Lee and Jeon, 2024). Heightened social self-efficacy may increase an individual’s confidence in managing emotional conflicts, thereby mitigating the impact of negative emotions. Secondly, social support plays a critical role in coping with stress and negative emotions. Research indicates that social support can act as a buffer against emotional distress, alleviating depressive symptoms (Lee and Jeon, 2024). For college students with subclinical depression, who may experience greater difficulty in acquiring and maintaining supportive social relationships, the positive interactions facilitated by social rewards could be particularly significant.

A distinct pattern emerged regarding social reward hierarchy effects. During emotional conflict trials, reaction times under class-level praise were significantly longer than other conditions for both groups, likely due to heightened evaluation pressure in immediate social circles—a phenomenon amplified by collectivistic cultural norms emphasizing “face” preservation. Conversely, in congruent trials, university-level praise elicited longer reaction times, reflecting the high value placed on institutional recognition within Chinese academic culture. This demonstrates that social reward value and cognitive demand interact to shape resource allocation similarly across groups, despite baseline differences in depressive tendencies. The findings underscore the culturally embedded motivational power of social rewards in modulating cognitive-emotional processing.

The dissociation between reward types underscores the importance of tailored interventions. Monetary rewards with optimal magnitude may enhance low-level perceptual tasks, while social recognition might better support complex socio-emotional regulation. These findings advocate for reward-sensitivity-informed approaches that consider both reward type and magnitude in cognitive-affective interventions for subclinical depression. Additionally, the cultural embeddedness of reward perception highlights the need for culturally adapted strategies in educational and clinical settings.

This study had several limitations, including its focus on a specific age group and the use of laboratory-based rewards. Although both reward types significantly modulated emotional and behavioral responses, their underlying neural mechanisms appear distinct (Anderson, 2016; Li et al., 2020; Toobaei et al., 2025). Future research should examine these mechanisms across diverse populations, incorporate neural imaging to elucidate underlying circuits, and explore longitudinal interventions that systematically vary reward types and magnitudes to refine personalized approaches.

### 4.4 Educational implications

These findings offer practical guidance for educational support. For students with depressive tendencies, high-value, tangible incentives may effectively boost engagement in learning tasks. Educational approaches could leverage this by incorporating clear, achievement-contingent rewards. Furthermore, since these students showed specific sensitivity to class-level (proximal) rather than school-level (distal) praise, feedback from close peers or teachers may be more motivating. Tailoring feedback to be more immediate and socially proximate could enhance its efficacy. Finally, their relative difficulty with emotionally congruent information suggests that simplifying emotional valence in instructional materials may reduce cognitive load and improve learning efficiency.

## 5 Conclusion

In summary, this study demonstrates that individuals with subclinical depression exhibit altered emotional conflict processing, which is differentially modulated by monetary and social reward gradients. For monetary rewards, a nonlinear threshold effect is observed, where improvements in performance (e.g., accuracy) reach a plateau after a specific reward magnitude, with no significant gains beyond that point. In contrast, social rewards exert a more uniform influence across groups, modulating performance in a similar pattern for both subclinical depression and healthy control participants without exhibiting a clear saturating threshold. These findings suggest that while basic cognitive-affective deficits exist in subclinical depression, motivational pathways remain accessible but are reward-type dependent. These findings suggest that while basic cognitive-affective deficits exist in subclinical depression, motivational pathways remain accessible but are reward-type dependent. Specifically, high-magnitude monetary incentives may partially normalize response speed in simple tasks, whereas social rewards engage broader cognitive-control networks that mitigate conflict-processing impairments. These results underscore the importance of incorporating reward-sensitivity gradients into the cognitive-affective profiling of subclinical populations. Future studies should leverage neuroimaging techniques to delineate the neural circuits underlying these dissociable effects and explore their translational potential for targeted behavioral interventions. Furthermore, while our exploratory analyses suggested no significant confounding effect of gender, future research should intentionally balance gender across groups and include it as a key factor to systematically examine its potential moderating role in reward-motivated emotional processing. Finally, it is important to note that our experimental design manipulated reward magnitude but did not directly assess reward efficacy (i.e., the subjective value or ‘wanting’ associated with a reward). Future studies could therefore employ paradigms that dissociate these components to better understand their unique contributions to motivational deficits in depression.

## Data Availability

The original contributions presented in the study are included in the article/supplementary material, further inquiries can be directed to the corresponding authors.

## References

[ref1] Ait Oumeziane B. Jones O. Foti D. (2019). Neural sensitivity to social and monetary reward in depression: clarifying general and domain-specific deficits. Front. Behav. Neurosci. 13:199. doi: 10.3389/fnbeh.2019.00199, 31649515 PMC6794449

[ref2] Anderson B. A. (2016). Social reward shapes attentional biases. Cogn. Neurosci. 7, 30–36. doi: 10.1080/17588928.2015.1047823, 25941868 PMC4654995

[ref3] Auerbach R. P. Mortier P. Bruffaerts R. Alonso J. Benjet C. Cuijpers P. . (2018). WHO world mental health surveys international college student project: prevalence and distribution of mental disorders. J. Abnorm. Psychol. 127, 623–638. doi: 10.1037/abn0000362, 30211576 PMC6193834

[ref4] Ba J. X. Wang L. J. (2023). Sensitivity to social and monetary rewards in individuals with depressive tendencies: the role of reward probability. J. Psychol. Sci. 46, 1220–1227. doi: 10.16719/j.cnki.1671-6981.20230524

[ref5] Başgöze Z. Gönül A. S. Baskak B. Gökçay D. (2015). Valence-based word-face Stroop task reveals differential emotional interference in patients with major depression. Psychiatry Res 229, 960–967. doi: 10.1016/j.psychres.2015.05.099 26272019

[ref6] Beblo T. Dehn L. B. (2019). “Clinical characteristics of emotional-cognitive dysfunction in major depressive disorder” in Cognitive dimensions of major depressive disorder (Oxford: Oxford University Press), 87–99.

[ref7] Beck A. T. Steer R. A. Ball R. Ranieri W. F. (1996). Comparison of Beck depression inventories-IA and-II in psychiatric outpatients. J. Pers. Assess. 67, 588–597. doi: 10.1207/s15327752jpa6703_13, 8991972

[ref8] Bijleveld E. van Breukelen F. N. de Segovia Vicente D. Schutter D. J. (2023). Mapping the dose–response relationship between monetary reward and cognitive performance. Motiv. Sci. 9, 205–215. doi: 10.1037/mot0000299

[ref9] Bob P. Susta M. Gregusova A. Jasova D. (2009). Dissociation, cognitive conflict and nonlinear patterns of heart rate dynamics in patients with unipolar depression. Prog. Neuro-Psychopharmacol. Biol. Psychiatry 33, 141–145. doi: 10.1016/j.pnpbp.2008.11.005, 19041359

[ref10] Botvinick M. M. Braver T. S. Barch D. M. Carter C. S. Cohen J. D. (2001). Conflict monitoring and cognitive control. Psychol. Rev. 108, 624–652. doi: 10.1037/0033-295x.108.3.624, 11488380

[ref22] CAS Institute of Psychology. (2025). Mental health blue book: report on the development of Chinese national mental health (2023–2024). Beijing, China: Institute of Psychology, Chinese Academy of Sciences (CAS).

[ref11] Chen F. Lian J. Zhang G. Guo C. (2022). Semantics-prosody Stroop effect on English emotion word processing in Chinese college students with trait depression. Front. Psych. 13:889476. doi: 10.3389/fpsyt.2022.889476 PMC9207235 35733799

[ref12] du Rocher A. R. Pickering A. D. (2024). Social interaction anxiety, social phobia, and cognitive control: controlled reactions to facial affect during an emotional face flanker task. Curr. Psychol 43, 4129–4141. doi: 10.1007/s12144-023-04624-y

[ref13] Etkin A. Egner T. Peraza D. M. Kandel E. R. Hirsch J. (2006). Resolving emotional conflict: a role for the rostral anterior cingulate cortex in modulating activity in the amygdala. Neuron 51, 871–882. doi: 10.1016/j.neuron.2006.07.029, 16982430

[ref14] Fan L. Xu Q. Wang X. Xu F. Yang Y. Lu Z. (2018). The automatic activation of emotion words measured using the emotional face-word Stroop task in late Chinese–English bilinguals. Cognit. Emot. 32, 315–324. doi: 10.1080/02699931.2017.1303451, 28332423

[ref15] Fang Z. Lynn E. Huc M. Fogel S. Knott V. J. Jaworska N. (2022). Simultaneous EEG+ fMRI study of brain activity during an emotional Stroop task in individuals in remission from depression. Cortex 155, 237–250. doi: 10.1016/j.cortex.2022.07.010, 36041320

[ref16] Gong X. Huang Y. X. Wang Y. Luo Y. J. (2011). Revision of the Chinese facial affective picture system. Chin. Ment. Health J. 25, 40–46. doi: 10.3969/j.issn.1000-6729.2011.01.011

[ref17] Guo Y. Huang X. Li Z. Li W. Shi B. Cui Y. . (2023). Aberrant reward dynamics in depression with anticipatory anhedonia. Clin. Neurophysiol. 154, 34–42. doi: 10.1016/j.clinph.2023.05.014, 37541075

[ref18] Guo Z. Wu X. Liu J. Yao L. Hu B. (2018). Altered electroencephalography functional connectivity in depression during the emotional face-word Stroop task. J. Neural Eng. 15:056014. doi: 10.1088/1741-2552/aacdbb, 29923500

[ref19] Haas B. W. Omura K. Constable R. T. Canli T. (2006). Interference produced by emotional conflict associated with anterior cingulate activation. Cogn. Affect. Behav. Neurosci. 6, 152–156. doi: 10.3758/cabn.6.2.152 17007235

[ref20] Hartlage S. Arduino K. Alloy L. B. (1998). Depressive personality characteristics: state dependent concomitants of depressive disorder and traits independent of current depression. J. Abnorm. Psychol. 107, 349–354. doi: 10.1037/0021-843x.107.2.349, 9604564

[ref21] Hu Z. G. Liu H. Y. Zhang X. X. (2008). Emotional conflict: a new research topic. Adv. Psychol. Sci. 16, 692–698.

[ref23] Jia Y. Cui L. Pollmann S. Wei P. (2021). The interactive effects of reward expectation and emotional interference on cognitive conflict control: an ERP study. Physiol. Behav. 234:113369. doi: 10.1016/j.physbeh.2021.113369, 33636632

[ref24] Klawohn J. Joyner K. Santopetro N. Brush C. Hajcak G. (2022). Depression reduces neural correlates of reward salience with increasing effort over the course of the progressive ratio task. J Affect Disord 307, 294–300. doi: 10.1016/j.jad.2022.03.051 35339572

[ref25] Kou M. Zhang H. Lv Y. Luo W. (2022). The effects of depression tendency and social comparison on adolescent self-evaluation. Neuropsychologia 170:108236. doi: 10.1016/j.neuropsychologia.2022.108236, 35405187

[ref26] Lakshmi P. M. Kishore M. T. Roopesh B. N. Jacob P. Rusanov D. Hallford D. J. (2023). Future thinking and anticipatory pleasure in adolescents with major depression: association with depression symptoms and executive functions. Clin. Child Psychol. Psychiatry 29, 526–539. doi: 10.1177/13591045231205004, 37807910

[ref27] Lee K.-Y. Jeon S.-E. (2024). The effect of mentalization and emotional clarity on depression among university students: the moderated mediating effect of social support. J. Korean Assn. Learn. Cent. Curric. Instr 24, 279–291. doi: 10.22251/jlcci.2024.24.11.279

[ref28] Li J. Liu L. Sun Y. Fan W. Li M. Zhong Y. (2020). Exposure to money modulates neural responses to outcome evaluations involving social reward. Soc. Cogn. Affect. Neurosci. 15, 111–121. doi: 10.1093/scan/nsaa019, 32064532 PMC7171377

[ref29] Liu W. H. Roiser J. P. Wang L. Z. Zhu Y. H. Huang J. Neumann D. L. . (2015). Anhedonia is associated with blunted reward sensitivity in first-degree relatives of patients with major depression. J. Affect. Disord. 190, 640–648. doi: 10.1016/j.jad.2015.10.050, 26590511 PMC5330646

[ref30] Loeffler L. A. K. Satterthwaite T. D. Habel U. Schneider F. Radke S. Derntl B. (2019). Attention control and its emotion-specific association with cognitive emotion regulation in depression. Brain Imaging Behav. 13, 1766–1779. doi: 10.1007/s11682-019-00174-9, 31414234

[ref31] Maunsell J. H. R. (2004). Neuronal representations of cognitive state: reward or attention? Trends Cogn. Sci. 8, 261–265. doi: 10.1016/S1364-6613(04)00102-0, 15165551

[ref32] Nie Y. Pan T. He J. Li Y. (2025). Blunted neural response to real-life social reward anticipation in internet gaming disorder: an event-related potential study. Int. J. Psychophysiol. 207:112479. doi: 10.1016/j.ijpsycho.2024.112479, 39637947

[ref33] Padmala S. Pessoa L. (2011). Reward reduces conflict by enhancing attentional control and biasing visual cortical processing. J. Cogn. Neurosci. 23, 3419–3432. doi: 10.1162/jocn_a_00011, 21452938 PMC3175266

[ref34] Pinho L. G. Engström M. Schneider B. C. Fonseca C. Lindberg M. Schröder J. . (2025). Symptoms of anxiety and depression among health and social science students: a multicenter study. Heliyon 11:e41957. doi: 10.1016/j.heliyon.2025.e41957, 39897836 PMC11786828

[ref35] Ros L. Satorres E. Fernandez-Aguilar L. Delhom I. Lopez-Torres J. Latorre J. M. . (2023). Differential effects of faces and words in cognitive control in older adults with and without major depressive disorder: an emotional Stroop task study. Appl Neuropsychol Adult 30, 239–248. doi: 10.1080/23279095.2021.1927037, 34137651

[ref36] Rosell-Negre P. Bustamante J. C. Fuentes-Claramonte P. Costumero V. Benabarre S. Barrós-Loscertales A. (2017). Monetary reward magnitude effects on behavior and brain function during goal-directed behavior. Brain Imaging Behav. 11, 1037–1049. doi: 10.1007/s11682-016-9577-7, 27473167

[ref37] Rutherford A. V. McDougle S. D. Joormann J. (2023). Don’t [ruminate], be happy: a cognitive perspective linking depression and anhedonia. Clin. Psychol. Rev. 101:102255. doi: 10.1016/j.cpr.2023.102255, 36871425

[ref38] Sankar A. Yttredahl A. A. Fourcade E. W. Mickey B. J. Love T. M. Langenecker S. A. . (2019). Dissociable neural responses to monetary and social gain and loss in women with major depressive disorder. Front. Behav. Neurosci. 13:149. doi: 10.3389/fnbeh.2019.00149 31354443 PMC6637282

[ref39] Shenhav A. (2024). The affective gradient hypothesis: an affect-centered account of motivated behavior. Trends Cogn. Sci. 28, 1089–1104. doi: 10.1016/j.tics.2024.08.003, 39322489 PMC11620945

[ref40] Tan X. Zhang J. Wu C. Y. Du J. Kong J. H. (2010). Characteristics of depressive tendencies in college students and their psychological intervention with traditional Chinese medicine. Jilin J Tradit Chin Med 30, 741–744. doi: 10.13463/j.cnki.jlzyy.2010.09.042

[ref41] Toobaei M. Taghavi M. Jobson L. (2025). The interactive role of emotion and expected efficacy and reward in improving cognitive control in patients with depression. BMC Psychiatry 25:406. doi: 10.1186/s12888-025-06847-8 40259301 PMC12010535

[ref42] Wang D. Liu T. Shi J. (2017). Development of monetary and social reward processes. Sci. Rep. 7:11128. doi: 10.1038/s41598-017-11558-6, 28894231 PMC5594021

[ref43] Wingenfeld K. Riedesel K. Petrovic Z. Philippsen C. Meyer B. Rose M. . (2011). Impact of childhood trauma, alexithymia, dissociation, and emotion suppression on emotional Stroop task. J. Psychosom. Res. 70, 53–58. doi: 10.1016/j.jpsychores.2010.06.003, 21193101

[ref44] Wongtongkam N. (2019). Influence of coping, self-esteem and social support on undergraduate students’ emotional distress. Health Educ. 119, 187–201. doi: 10.1108/he-01-2019-0001

[ref45] Xue S. Wang S. Kong X. Qiu J. (2017). Abnormal neural basis of emotional conflict control in treatment-resistant depression: an event-related potential study. Clin. EEG Neurosci. 48, 103–110. doi: 10.1177/1550059416631658, 26892803

[ref46] Yang W. H. Xiong G. (2016). Validity and cut-off scores of commonly used depression scales for screening depressive disorders in Chinese adolescents. Chin. J. Clin. Psychol. 24, 1010–1015. doi: 10.16128/j.cnki.1005-3611.2016.06.011

[ref47] Zhang D. D. Wang J. Zhao J. Chen S. M. Huang Y. L. Gao Q. F. (2020). The influence of depressive tendencies on cooperation: a hyperscanning functional near-infrared spectroscopy study. Acta Psychol. Sin. 52, 609–622. doi: 10.3724/SP.J.1041.2020.00609

[ref48] Zhu X.-r. Zhang H.-j. Wu T.-t. Luo W.-b. Luo Y.-j. (2010). Emotional conflict occurs at an early stage: evidence from the emotional face-word Stroop task. Neurosci. Lett. 478, 1–4. doi: 10.1016/j.neulet.2010.04.036, 20417689

